# Validation of the Child Post-Traumatic Cognitions Inventory in Korean survivors of sexual violence

**DOI:** 10.1186/s13034-018-0235-2

**Published:** 2018-06-18

**Authors:** Han Byul Lee, Kyoung Min Shin, Young Ki Chung, Namhee Kim, Yee Jin Shin, Un-Sun Chung, Seung Min Bae, Minha Hong, Hyoung Yoon Chang

**Affiliations:** 1 Sunflower Center of Southern Gyeonggi for Women and Children Victims of Violence, Suwon, Republic of Korea; 2 Hanyang Cyber University, Seoul, Republic of Korea; 3 Department of Psychiatry and Behavioral Sciences, Ajou University School of Medicine, 164 World Cup-ro, Yeongtong-gu, Suwon, Suwon-si 16409 Republic of Korea; 4 Center for Traumatic Stress, Ajou University Medical Center, Suwon, Republic of Korea; 5 Yonsei University College of Medicine, Seoul, Republic of Korea; 6 Kyungpook National University Hospital, Daegu, Republic of Korea; 7 Gil Hospital, Gachon University College of Medicine, Incheon, Republic of Korea; 8 Myongji Hospital, Seonam University College of Medicine, Goyang, Republic of Korea

**Keywords:** Child Post-traumatic Cognitions Inventory, Post-traumatic cognitions, Child sexual abuse, Sexual violence, Psychometry

## Abstract

**Background:**

Dysfunctional cognitions related to trauma is an important factor in the development and maintenance of post-traumatic stress disorder symptoms in children and adolescents. The Child Post-traumatic Cognitions Inventory (CPTCI) assesses such cognitions about trauma. We investigated the psychometric properties of the Korean version of CPTCI and its short form by surveying child and adolescent survivors of sexual violence.

**Methods:**

Children and adolescents aged 7–16 years (*N* = 237, *M*_age_ = 12.6, *SD* = 2.3, 222 [93.7%] were female) who were exposed to sexual violence were included in this survey. We assessed the factor structure, internal consistency, and validity of the CPTCI and its short form through data analysis.

**Results:**

Confirmatory factor analysis results supported the two-factor model presented in the original study. The total scale, its subscales, and the short form had good internal consistency (Cronbach’s α = .96 for total scale and .91–.95 for the other scales). The CPTCI showed high correlations with scales measuring post-traumatic stress symptoms (*r *= .77–.80), anxiety (*r* = .69–.71), and depression (*r* = .74–.77); the correlation with post-traumatic stress symptoms was the highest. The differences in CPTCI scores per post-traumatic stress symptom levels were significant (all *p *< .001) Sex differences in CPTCI scores were not significant (*p *> .05 for all comparisons); however, the scores exhibited differences per age group (all *p* < .001).

**Conclusions:**

The results indicate that the Korean version of the CPTCI is a valid and reliable scale; therefore, it may be a valuable tool for assessing maladaptive cognitions related to trauma in research and clinical settings.

## Background

Recently, researchers have shown an increased interest in individual differences in how people respond to trauma [1, 2]. Some people experience minimal emotional distress and show only minor and transient disruptions in their ability to function, while others suffer from more intense pain that lasts longer [1]. Researchers have addressed post-traumatic cognitions as one of the factors influencing the severity and persistence of pathological responses to trauma [3–6]. Traumatic events significantly alter survivors’ cognitions and beliefs about themselves, the world, and their future, possibly leading to negative emotional responses and maladaptive actions, which in turn contributes to the development and maintenance of PTSD [4, 5]. The importance of such trauma-related cognitions is reflected in the fifth edition of the Diagnostic and Statistical Manual of Mental Disorders’ [7] (DSM-5; American Psychiatric Association 2013) revised diagnostic criteria for PTSD. One of the symptom clusters listed among the DSM-5′s diagnostic criteria for PTSD is “negative alterations in cognitions and mood,” which includes criterion D2 (“persistent and exaggerated negative beliefs or expectations about oneself, others, or the world [e.g., ‘I am bad’ and ‘no one can be trusted’])” and D3(“persistent, distorted cognitions about the cause or consequences of the traumatic event(s) that lead the individual to blame himself/herself or others.”) (p. 272). Such changes in diagnostic criteria emphasize the importance of assessment of trauma-related cognitions.

Considering the need for a valid and reliable instrument to assess trauma-related cognitions, Foa et al. [6] developed the Post-traumatic Cognitions Inventory (PTCI). This inventory consists of 33 items that comprise three factors: “negative cognitions about the self,” “negative cognitions about the world,” and “self-blame.” The inventory was translated and tested on diverse samples in countries such as Germany [8], the Netherlands [9], Korea [10], and Taiwan [11], where its factor structure was repeatedly verified, and its reliability and validity were confirmed. In these studies, it was reported that certain characteristics like sex, type of trauma experienced, and cultural background could affect PTCI scores and its psychometric properties.

Various studies have shown that cognitive models of PTSD can be extended to children and adolescents; however, they have also indicated the need to consider developmental aspects [12–14]. Therefore, Meiser-Stedman et al. [15] developed the Child Post-traumatic Cognitions Inventory (CPTCI) to assess the post-traumatic cognitions of children and adolescents. They made age-appropriate modifications to the PTCI items, and added some items based on a cognitive model of PTSD to construct an initial 41-item questionnaire that was used with a community sample comprised 223 children and adolescents. Based on the survey results, the researchers performed item reduction to arrive at the final 25-item questionnaire and validate it in two other sets of samples. Unlike the adult version, the CPTCI comprises only two subscales. First, the “permanent and disturbing change” subscale (CPTCI-PC) comprises 13 items and focuses on the negative effects that a frightening event has on a child and the child’s perception of the future. Second, the “fragile person in a scary world” subscale (CPTCI-SW) comprises 12 items and assesses the child’s sense of vulnerability and perception of the world and other people as threatening. One of the factors of the PTCI, “self-blame,” is not included in the CPTCI.

The CPTCI turns out to be a valid and reliable measure regarding multiple criteria, benefits from being standardized within a large population of children and adolescents [15]. Based on the theoretical model of PTSD, it has been proposed that the cognitive therapy of PTSD should target post-traumatic cognitions, and studies treating post-traumatic cognitions as a mediator of therapeutic change are being conducted using CPTCI [16–20]. Furthermore, a recently published study updated the CPTCI and evaluated its utility and psychometric properties, providing additional information on the test–retest reliability of CPTCI, as well as suggesting a short form of CPTCI and cutoff points in CPTCI scores for clinical use [21].

The CPTCI has been translated into several languages, and different versions have been validated and their psychometric properties have been reported in Germany [22], the Netherlands [17], Brazil [23] and Taiwan [24]. Previous studies generally report adequate levels of reliability and validity. Moreover, in all these samples the two-factor structure emerged as the best solution. These studies, however, showed that the original two-factor structure of CPTCI exhibits unsatisfactory model fit indices that do not meet the widely accepted criteria [17, 21, 22, 24, 25]. Authors attribute these discrepancies to sample characteristics and cultural differences. In the original study, most of the participants were exposed to traumatic events that did not last for longer than a few minutes and affected few people (e.g., motor vehicle accidents). In contrast, Taiwanese sample predominantly comprises natural disaster survivors. Meanwhile, the majority of children participated in the Brazilian CPTCI study experienced multiple traumas, such as ongoing physical or sexual abuse. The Dutch version and The German version of the CPTCI were also validated in the samples including survivors of interpersonal violence.

To address the issue, the Brazilian version used an exploratory factor analysis to derive a new two-factor model with items that were different from those of the extant two-factor model [23]. In the Chinese version of the CPTCI developed in Taiwan, researchers revised the original PTCI by deleting five items based on the results of confirmatory factor analysis (CFA). Both methods result in theoretically less sound models because the models were modified based on the results of the analysis. The models neither have enough empirical grounds. Therefore, the models need to replicate, in new sets of samples [26].

Sexual violence is a type of trauma that leads to severe psychological aftereffects. Sexual assault and sexual violence jointly make up the second largest share of traumas causing PTSD worldwide [27]. Especially, Child sexual abuse is associated with numerous adverse sequelae during childhood including depression, anxiety, behavioral problems, and post-traumatic stress disorder (PTSD), and is also correlated with an increased risk for mental health problems in adulthood [28, 29]. Several studies have shown that post-traumatic cognitions in survivors of sexual violence play a significant role in how they adapt afterwards [30, 31]. Some studies utilizing PTCI reported that a higher proportion of sexual violence survivors had maladaptive post-traumatic cognitions and beliefs as compared to survivors of other types of trauma [6, 11]. Moreover, since it is now well established that child sexual abuse survivors benefit from TF-CBT targeting maladaptive post-traumatic cognitions, assessment of post-traumatic cognitions in these populations is crucial for intervention [32, 33]. To date, however, the CPTCI has not been tested on a sample consisting solely of sexual violence survivors to determine its psychometric properties.

Consequently, this study has the following goals. First, we aimed to verify the factor structure of the CPTCI regarding child and adolescent survivors of sexual violence in Korea. Specifically, we sought to determine whether the original two-factor structure derived in the process of developing the scale could be used without adapting it for cultural differences or types of trauma. Second, we aimed to determine the convergent validity and discriminant validity of the CPTCI in comparison with scales that measure the severity of trauma symptoms, anxiety, and depression. Third, we examined the factor structure, reliability, and validity of the short form of the CPTCI (CPTCI-S) [21].

## Methods

### Participants

Children and adolescents (*N* = 237) aged 7–16 years who visited support centers for sexual assault survivors to receive medical, investigative, and counseling support after being exposed to sexual violence were included in the analysis. The sample was collected from four sexual assault victim support centers located across Korea from 2014 to 2016. Demographic variables and trauma-related information are shown in Table 1.

**Table 1:** Demographic characteristics and trauma-related Information

Variable	Total sample (*N *= 237)	CPTCI total		CRIES		TSCC-PT		CDI		RCMAS	
	*n* (%)	*m*	*sd*	*m*	*sd*	*m*	*sd*	*m*	*sd*	*m*	*sd*
Sex											
Male	15 (6.3)	52.73	17.64	31.87	12.36	12.57	8.17	18.33	10.33	16.07	8.40
Female	222 (93.7)	52.14	19.62	32.18	17.23	11,70	8.23	17.62	9.61	20.98	8.41
Age groups											
8–11	76 (32.1)	45.17	17.84	27.08	16.09	9.72	7,49	13.83	8.62	17.76	9.27
12–14	106 (44.7)	53.16	18.82	33.49	16.66	12.43	8.59	18.80	9.11	22.08	7.64
15–16	55 (23.1)	59.96	19.81	36.53	17.19	13.37	8.00	20.73	10.43	21.96	7.97
Type of trauma											
Rape	94 (39.7)	56.31	19.22	33.78	16.25	12.63	8.40	20.32	9.20	21.90	7.96
Sexual abuse other than rape	143 (60.3)	49.46	19.21	31.10	17.35	11.21	8.07	15.94	9.56	19.85	8.73
Time since trauma											
Less than 1 week	98 (41.4)	51.28	19.13	32.28	17.65	11.66	8.29	18.19	10.35	20.82	8.24
1 week–1 month	40 (16.9)	56.25	20.46	35.90	16.91	13.23	8.62	18.65	10.22	21.78	8.76
1–3 months	20 (8.4)	48.90	16.51	27.75	14.94	10.63	7.07	15.75	8.47	19.25	8.07
3 months or more	72 (30.4)	52.58	19.82	31.40	16.30	11.25	8.16	16.83	8.50	20.28	8.89
Unspecified	7 (3.0)	50.17	23.84	34.33	16.17	13.00	9.72	19.00	12.43	21.17	8.80
Region											
Suwon	164 (69.2)	51.66	19.70	32.96	16.71	11.92	8.19	17.64	9.92	20.71	8.61
Seongnam	28 (11.8)	54.46	19.49	34.43	16.75	12.57	8.09	17.57	8.02	20.68	8.30
Goyang	27 (11.4)	55.67	17.03	32.38	14.30	12.81	8.44	19.23	9.25	21.00	7.52
Jeju	18 (7.6)	48.06	21.09	21.06	20.00	7.33	7.39	15.83	10.20	19.78	9.47

*CPTCI total* Total Score of Korean version of Child Post-Traumatic Cognitions Inventory, *CRIES* Children’s Revised Impact of Event Scale, *TSCC-PT* Post-traumatic stress Subscale of the Traumatic Symptom Checklist for Children, *CAPS* Children’s Attributions and Perceptions Scale, *CDI* Children’s Depression Inventory, *RCMAS* Revised Children’s Manifest Anxiety Scale

### Procedure

Questionnaire results were obtained with the consent of the survivors themselves and their guardians who provided consent for the collection and use of the data for research purposes. The questionnaire was completed with paper and pencil by the survivors and included the CPTCI, CRIES, TSCC, CDI, and RCMAS. The questionnaires that were submitted at each of the support centers were collected at a single center along with basic information on the survivors and details about the traumatic incidents they had experienced. All the procedures conducted by this study were reported to and approved by the Institutional Review Board of the Ajou University Medical Center (IRB number: SBR-SUR-17-041).

### Measures

#### CPTCI

The CPTCI is a self-report questionnaire consisting of 25 items that is designed to assess dysfunctional trauma-related cognitions in children and adolescents [15]. Each item is rated on a four-point Likert scale: “*do not agree at all*” (1 point), “*do not agree a bit*” (2 points), “*agree a bit*” (3 points), and “*agree a lot*” (4 points). Two factors were confirmed in the process of developing the scale: CPTCI-PC and CPTCI-SW. CPTCI-PC has 13 items and CPTCI-SW has 12 items; the scores each are calculated along with the total score. A higher score indicates greater dysfunction in trauma-related cognitions. The reliability and the validity of the CPTCI total score and its subscales were reported to be adequate in the original paper. In 2016, the researchers of the original paper developed the CPTCI-S [21]. The short form comprises 6 items from the CPTCI-PC subscale and 4 items from the CPTCI-SW subscale. Items were selected on the basis of factor loadings and relationships with the CPTCI total score as well as a PTSD diagnosis. The 2016 study found that the CPTCI-S had excellent psychometric properties. As for the Korean version of the CPTCI, the second author of this study (KMS) received permission from one of the CPTCI authors (i.e. Meiser-Stedman, R.), to translate the CPTCI items into Korean. Then, the corresponding author (HYC), a child and adolescent psychiatrist and bilingual speaker of Korean and English, reviewed the translated items. Total score of the CPTCI ranges 25–100.

#### Children’s Revised Impact of Event Scale (CRIES)

The CRIES is used to assess children who have been exposed to traumatic events and are at risk of suffering from PTSD [34]. The CRIES comprises 13 items measuring various PTSD symptoms like intrusion, avoidance, and hyperarousal. Each item is rated on a four-point Likert scale (0 = “*not at all*,” 1 = “*rarely*,” 3 = “*sometimes*,” and 5 = “*often*”). The score for each item is summed to yield a total score; higher scores indicate greater severity of children’s post-traumatic stress response. We used the Korean version of the CRIES in this study [35]. The Korean version of the CRIES exhibited adequate levels of internal consistency (Cronbach’s α = .93 for the total scale) and both convergent and discriminant validity. The study proposed a cutoff of 26 to screen PTSD in children and adolescent. Total score of CRIES ranges 0–65.

#### Trauma Symptom Checklist for Children (TSCC)

The TSCC is a self-report assessment scale that was designed by Briere [36]. It includes two validity scales measuring under-response and hyper-response, along with six clinical scales measuring anxiety, depression, anger, post-traumatic stress symptoms, dissociation (two subscales on overt dissociation and fantasy), and sexual concerns (two subscales on sexual preoccupation and sexual distress). In this study, we use post-traumatic stress subscale to measure post-traumatic symptoms severity. Post-traumatic stress subscale comprises 10 items rated on a four-point scale ranging from 0 (“*never*”) to 3 (“*almost all of the time*”). We used a version of the scale translated by Son and colleagues (2007), which reported an internal consistency of α = .97.

#### Revised Children’s Manifest Anxiety Scale (RCMAS)

The RCMAS was developed by Castenada, McCandlless, and Palermo (1956) to measure the manifest anxiety of children and adolescents, and revised and supplemented by Reynolds and Richmond [37]. It consists of 37 items addressing anxiety, asking the child to answer *yes/no* on how the child thinks and feels about oneself. We used the Korean version of the RCMAS, which was translated by Choe et al. [38] and has an adequate level of internal consistency (α = .81). Total score of the RCMAS ranges from 0 to 37.

#### Children’s Depression Inventory (CDI)

To test the convergent validity of the CPTCI, we used the CDI, which measures depression in children. The CDI was devised by Kovacs [39] to assess the depression of school-aged children and adolescents. It comprises 27 items, and each item consists of three statements. The statement that most closely matches their mood over the past 2 weeks is chosen by respondents. Total score of the CDI ranges from 0 to 54. The Korean version of the CDI has adequate reliability and validity (i.e., α = .76.).

### Statistical analysis

The data were analyzed as follows. First, a confirmatory factor analysis was performed on the sample using AMOS 18.0 to assess the factor structure of the Korean version of the CPTCI [40]. To determine the valid factor structure through model comparisons, three models of the full scale were tested. The first model is the two-factor one presented in the original paper using the CPTCI-PC and CPTCI-SW subscales, and each item was restricted to load on just one fixed factor. The modified two-factor model for the Brazilian version of CPTCI was presented in Lobo et al. [23]; like the original model, it consists of two factors: CPTCI-PC and CPTCI-SW. However, these factors comprise 14 and 11 items, respectively in the Brazilian model, and the items included in each factor are also different from those in the original model. In the one-factor model, all 25 items were made to load on one factor.

Besides, we tested 20-item model which Taiwanese researchers have proposed. Removed items are item number 3, 8, 12, 14 and 25. Other items are loaded onto the same factor as the original version.

Aside from these factor models, we also tested the factor structure of the 10-item CPTCI-S. In the CPTCI-S, six items load on the CPTCI-PC and four items load on the CPTCI-SW. To compare different models of the CPTCI-S, a one-factor model that accounts for all items for one factor was tested here as well.

The χ^2^ test results and fit indices for each model were compared. χ^2^ index is very sensitive to sample size, making it highly likely to commit the error of dismissing the null hypothesis. Therefore, it is necessary to consider the χ^2^ index in conjunction with other goodness-of-fit indices [25]. Based on the criteria proposed in earlier studies, we set the root mean square error of approximation (RMSEA) < .08 and comparative fit index (CFI) and Tucker–Lewis index (TLI) at > .90 as the criteria for judging goodness of model fit [25, 41]. Because the scores were not normally distributed, the method of maximum-likelihood estimation was applied using the Bollen-Stine bootstrap procedure. To assess the internal consistency of the scale, Cronbach’s α values were computed for the full scale, the two subscales, and the CPTCI-S. Next, to assess convergent validity, Pearson correlation coefficients were calculated for the CPTCI, PTSD, anxiety, and depression scales. Then, to determine discriminant validity, we conducted an independent samples *t* test comparing a high PTSD-risk group and a low PTSD-risk group with respect to their CPTCI total scores and scores for the two subscales and the short form. The high PTSD-risk group and the low PTSD-risk group were classified based on the cutoff point of the CRIES (i.e., a score of 26 points) [35]. For differences in CPTCI scores that depend on demographic variables, we performed t-tests and an analysis of variance (ANOVA) per sex and three age groups (8–11-year-olds, 12–14-year-olds, and 15–16-year-olds). All statistical analyses were conducted using SPSS 18.0 [42] and AMOS 18.0 [40].

## Results

### Confirmatory factor analysis

A confirmatory factor analysis revealed a significant disparity between the model and the observed data regarding the original two-factor model for the full scale: χ^2^(274, *N *= 237) = 878.2, *p *< .001. Excluding the SRMR, the values indicate that the model’s goodness of fit falls short of the criteria set earlier (see Table 2). Lobo et al. (2015) two-factor model exhibited somewhat poorer fit in comparison with the original two-factor model, χ^2^(274, *N* = 237) = 908.0, as did the one-factor model, χ^2^(275, *N* = 237) = 981.8. Moreover, when the χ^2^ test was used to compare the one-factor model with the original two-factor model, it was found that Δχ^2^ = 103.6 with a significance level of *p *= .01. Liu and Chen’s (2015) 20-item two-factor model yielded better fit indices in CFI, TLI, RMSEA, and SRMR than the original 25-item model did. χ^2^(169, *N* = 237) = 528.1. The fit indices for the two-factor model of CPTCI-S (χ^2^(34, *N* = 237) = 106.7) revealed that all the fit indices except RMSEA showed good model fit; and the two-factor model fared much better than the one-factor model (Table 2). Although goodness-of-fit indices indicate that Liu and Chen’s (2015) 20-item model fits better the data than the original 25-item model, we agreed that there are several issues need to be addressed to use the 20-item model and decided to retain the all 25 items and use the data of full version of the CPTCI in the rest of the article. Backgrounds for the decision is discussed in the discussion section. The factor coefficients analyzed using the original two-factor model are shown in Table 3.

**Table 2:** Summary of results from confirmatory factor analyses

Model	*X* ^2^	*df*	CFI	TLI	RMSEA	90% CI	SRMR	Removed items
Cut-off criteria of the GoF	–	–	> .90	> .90	< .08		< .08	
CPTCI original 25-item version								
1. Original two-factor [15]	878.2	274	.858	.844	.097	.090–.104	.057	None
2. Modified two-factor [23]	908.0	274	.851	.837	.099	.092–.106	.058	None
3. One-factor	981.8	275	.834	.819	.104	.097–.111	.060	None
CPTCI 20-item version [24]	528.1	169	.896	.884	.095	.086–.104	.051	3, 8, 12, 14, 25
CPTCI-S [21]								
1. Original two-factor	106.7	34	.950	.933	.095	.075–.116	.038	1, 2, 3, 89, 11, 12, 13, 17, 18, 20, 22, 23, 24, 25
2. One-factor	114.0	35	.945	.929	.098	.078–.118	.039	

*GoF* goodness-of-Fi, *CFI* comparative fit index, *TLI* Tucker–Lewis index, *RMSEA* root mean square error of approximation, *CI* confidence interval, *SRMR* standardized root mean square error of approximation, *CPTCI* Child Post-traumatic Cognitions Inventory, *CPTCI-S* short form of the CPTCI

**Table 3:** Factor Loadings of Korean CPTCI

	Item	CPTCI-PC		CPTI-SW	
		Original	CPTCI-S	Original	CPTCI-S
4.	My reactions since the frightening event mean I have changed for the worse	.764	.857		
6.	My reactions since the frightening event mean something is seriously wrong with me	.823	.887		
8.	Not being able to get over all my fears means that I am a failure	.750			
13.	My reactions since the frightening event mean I will never get over it	.729			
14.	I used to be a happy person but now I am always sad	.707	.753		
16.	I will never be able to have normal feelings again	.771	.800		
17.	I’m scared that I’ll get so angry that I’ll break something or hurt someone	.740			
19.	My life has been destroyed by the frightening event	.796	.819		
20.	I feel like I am a different person since the frightening event	.768			
21.	My reactions since the frightening event show that I must be going crazy	.836	.838		
22.	Nothing good can happen to me anymore	.814			
23.	Something terrible will happen if I do not try to control my thoughts about the frightening event	.762			
24.	The frightening event has changed me forever	.792			
1.	Anyone could hurt me			.531	
2.	Everyone lets me down			.679	
3.	I am a coward			.565	
5.	I don’t trust people			.696	.776
7.	I am no good			.780	.808
9.	Small things upset me			.683	
10.	I can’t cope when things get tough			.758	.776
11.	I can’t stop bad things from happening to me			.792	
12.	I have to watch out for danger all the time			.458	
15.	Bad things always happen			.749	.778
18.	Life is not fair			.807	
25.	I have to be really careful because something bad could happen			.553	

*CPTCI* Child Post-traumatic Cognitions Inventory, *CPTCI-PC* permanent and disturbing change subscale of the CPTCI, *CPTCI-SW* fragile person in a scary world subscale of the CPTCI, *Original* original form of the CPTCI, *CPTCI-S* short form of the CPTCI

### Reliability

The Cronbach’s α for CPTCI was .96, showing that the scale was highly reliable. The internal consistency of the two subscales were .95 for CPTCI-PC and .91 for CPTCI-SW. The internal consistency of the CPTCI-S was .93. The correlations among the CPTCI total score and the scores for the two subscales as well as the short form were significant and strong (range = .85 to .98). The correlations are provided in Table 4.

**Table 4:** Correlations among the CPTCI and other study measures

		CPTCI total	CPTCI-PC	CPTCI-SW	CPTCI-S
1.	CPTCI total	–	.97	.96	.98
2.	CPTCI-PC		–	.85	.96
3.	CPTCI-SW			–	.91
4.	CPTCI-S				–
5.	CRIES				
	r	.80	.77	.78	.79
	rp	.61	.57	.50	.56
6.	TSCC-PT				
	r	.78	.74	.78	.77
	rp	.54	.48	.51	.51
7.	CDI	.76	.77	.77	.74
8.	RCMAS	.71	.73	.73	.69

*r* correlation coefficient, *rp* partial correlation coefficients between the CPTCI and other study measures controlling for CDI and RCMAS, *CPTCI total* Total Score of Korean version of Child Post-Traumatic Cognitions Inventory, *CPTCI-PC* Permanent and disturbing Change subscale of the CPTCI, *CPTCI-SW* fragile person in a scary world subscale of the CPTCI, *CPTCI-S* Short form of the CPTCI, *CRIES* Children’s Revised Impact of Event Scale, *TSCC-PT* post-traumatic stress subscale of the Traumatic Symptom Checklist for Children, *CAPS* Children’s Attributions and Perceptions Scale, *CDI* Children’s Depression Inventory, *RCMAS* Revised Children’s Manifest Anxiety Scale. All correlations *p *< .001

### Validity

To assess the convergent validity of the CPTCI, we computed its Pearson correlations with other self-report scales. The CPTCI exhibited significant correlations in all the measured values at the level of *p *< .001 with two scales for measuring post-traumatic stress symptoms: the CRIES and TSCC. The CPTCI’s total score, the scores for the two subscales, and the scores for the short form showed correlations ranging from .77 to .80 with the CRIES, and ranging from .74 to .78 with the TSCC-PT. Correlations between CPTCI scores and the CDI were high at .76 to .77, and correlations with the RCMAS were also higher than .7. However, these correlations were relatively low compared to those between the CPTCI and the two scales measuring PTSD symptoms. To verify that the correlations between CPTCI and PTSD symptoms are not an artifact arising from the correlations among trauma-related cognitions and depression and anxiety, partial correlations between CPTCI and PTSD scales were computed while controlling for CDI and RCMAS scores. The results showed that the correlations among CPTCI total score, CPTCI-PC, and CPTCI-SW remained strong. The partial correlation coefficients between CPTCI and CRIES scores ranged from .50 to .61, and those between CPTCI and TSCC-PT scores ranged from .48 to .54 (Table 4).When comparing CPTCI scores and PTSD symptoms severity by age groups, similar correlations were observed between CPTCI scores and PTSD symptom severity (.68–.85). Though the correlations coefficients tend to be the stronger in the older group(15–16 years old), they did not differed significantly per age group.

### Discriminant validity

Table 5 shows the results of comparing two groups considering differences in their CPTCI total score and scores for the subscales and the short form corresponding to differences in the severity of PTSD symptoms. The high PTSD-risk group (*n* = 152) and the low PTSD-risk group (n = 85) showed significant differences in various CPTCI scores (Table 5). All between groups differences were very large (all d’s > 1.92).

**Table 5:** Comparison of Korean CPTCI scores by sample characteristics

	CPTCI total					CPTCI-PC				CPTCI-SW				CPTCI-S			
	*n*	*m*	*sd*	*t* or *F*	*d* or *ε*^2^	*m*	*sd*	*t* or *F*	*d* or *ε*^2^	*m*	*sd*	*t* or *F*	*d* or *ε*^2^	*m*	*sd*	*t* or *F*	*d* or *ε*^2^
				***F***	***ε*** ^**2**^			***F***	***ε*** ^**2**^			***F***	***ε*** ^**2**^			***F***	***ε*** ^**2**^
Age groups^a^																	
8–11 (1)	76	45.17	17.84	10.19**	.087	21.03	9.08	8.95**	.077	24.42	9.36	9.38**	.080	17.04	7.18	10.35**	.089
12–14 (2)	106	53.16	18.82			24.53	10.53			28.63	9.16			20.39	8.30		
15–16 (3)	55	59.96	19.81			28.69	11.03			31.27	9.24			23.40	8.25		
–				***t***	***d***			***t***	***d***			***t***	***d***			***t***	***d***
+-Type of trauma																	
Rape	94	56.31	19.22	2.69*	.35	26.66	10.54	2.73*	.36	29.65	9.30	2.31	.32	21.85	8.04	2.83*	.38
Sexual abuse other than rape	143	49.46	19.21			22.88	10.33			26.74	9.60			18.79	8.21		
				***t***	***d***			***t***	***d***			***t***	***d***			***t***	***d***
Time since trauma																	
Less than 1 month	138	52.72	19.58	.36	.05	24.63	10.85	.43	.06	28.09	9.47	.25	.03	20.22	8.45	.37	.05
More than 1 month	99	51.78	19.12			24.02	10.31			27.76	9.55			19.80	8.11		
				***t***	***d***			***t***	***d***			***t***	***d***			***t***	***d***
PTSD symptoms																	
Higher	152	62.03	16.74	14.14**	2.07	29.41	9.72	15.99**	1.92	32.61	8.03	15.05**	1.92	24.06	7.32	13.37**	1.98
Lower	85	34.56	8.44			15.29	3.64			19.46	5.38			12.73	3.49		

*CPTCI* Child Post-traumatic Cognitions Inventory, *CPTCI-PC* permanent and disturbing change subscale of the CPTCI, *CPTCI-SW* fragile person in a scary world subscale of the CPTCI, *CPTCI-S* Short form of the CPTCI

* p < .01, ** p < .001

^a^Comparisons of the three groups by Sheffe post-test

### Differences by age group, phase and type of trauma

CPTCI scores by age group, phase following traumatic stressor and type of trauma are listed in Table 5. Based on the PTSD diagnostic criteria, acute/chronic groups were divided on a monthly basis. All the indices show that there were no significant differences in CPTCI scores between acute and chronic group; however, there were significant differences in CPTCI scores per age group as shown in the results of the ANOVA, CPTCI total score: *F*(2,234) = 10.19, *p *< .001; CPTCI-PC: *F*(2,234) = 8.95, *p* < .001; CPTCI-SW: *F*(2,234) = 9.38, *p* < .001; and CPTCI-S: *F*(2,234) = 9.38, *p* < .001. Scheffé’s post hoc test results showed that there were significant differences in all the scores except those for CPTCI-PC at the p = .05 level between 8 and 11-year-olds and 12- to 14-year-olds and between 8 and 11-year-olds and 15- to 16-year-olds. As for CPTCI-PC scores, the difference between 8 and 11-year-olds and 15- to 16-year-olds was significant. In all indices, an older age was accompanied by higher scores. Individuals were classified into two subgroups regarding types of sexual trauma they experienced. These subgroups differed on the CPTCI index scores, CPTCI total score: *t *= 2.69, *p *= .008; CPTCI-PC: *t *= 2.73, *p *= .007; CPTCI-SW: *t *= 2.31, *p *= .022; CPTCI-S: *t *= 2.83, *p *= .005; (Table 5).

## Discussion

We investigated the psychometric properties of the Korean version of the CPTCI by examining child and adolescent survivors of sexual violence in Korea. This study is the first to validate the CPTCI among Koreans and the first to apply the scale in a sample of survivors exposed to one specific type of trauma (i.e., sexual violence).

Our confirmatory factor analysis revealed that the original two-factor model has the best fit to data among the 25-item models subjected to comparison. Additionally, each of the two factors is loaded on all the items at appropriate levels in the factor matrix, which seems to support the two-factor model. Moreover, model comparison via the χ^2^ test showed that the original two-factor model was superior to the one-factor model [23]. Nevertheless, it was revealed that some model fit values for the original two-factor model fell short of the criteria set based on earlier studies.

The 20-item Chinese version showed better fit indices than the original version. This finding may have significant implications for understanding cultural effects on response to trauma. In this model, the items 3 (I am a coward), 12 (I have to watch out for danger all the time), and 25 (I have to be really careful because something bad could happen) were deleted because their standardized factor loadings were insufficient [24]. Researchers inferred that in Chinese culture, such cognitions of preparing for dangers are common in parenting and are internalized in children’s self-discipline. It is interesting to note that the current study also found that Item 3, 12 and 25 yielded relatively low factor loadings in CFA. A possible explanation for this similarity might be that Confucianism-based societal norms that East Asian societies have in common.

Nevertheless, we did not adopt the model for following reasons. First, although goodness-of-fit values of the model are better than those of the original model, they still fell short of the criteria set based on earlier studies, and are inferior to those of the CPTCI-S. Second, the version does not include one of the items which consist the CPTCI-S, making it difficult used along with the short form. Third, the item selection was based on the results of CFA in the study which it originates, which can be methodologically problematic. Last, utilizing the version which comprises different items from the original one would not allow the opportunity to compare research on the CPTCI across the regions.

The repetitive failures of different versions of the CPTCI to replicate the original factor structure seems to be related to the characteristic of different sample. In the original study, Researchers have raised the possibility that being at different stages of post-traumatic reactions may have an impact on factor structure and factor loadings. The participants in this study, in many cases, completed the questionnaire when they had visited the support center to receive crisis intervention immediately after their exposure to sexual violence. It is believed that trauma-related cognitions exhibited during the acute phase of traumatic stress may differ in kind and degree from cognitions exhibited after some passage of time when they have naturally recovered or become negatively distorted and consolidated [15, 43, 44].

Furthermore, the type of trauma experienced by the sample group in this study differs from that experienced by the samples in the original study. The original study used its scale on children who were exposed to a single traumatic event; more specifically, a traffic accident or a violent incident, and derived its factor structure from this basis. Therefore, negative cognitions related to physical injury and internal vulnerability could have become more salient. In contrast, this study was conducted with child and adolescent survivors of sexual violence, representing a mix of single, multiple, or complex trauma survivors. Other studies that have translated and validated the CPTCI, unlike the original study, included many participants who were exposed to continuous trauma like sexual violence and abuse. These studies have likewise reported that they could not confirm a good enough model fit for the original two-factor structure [17, 22, 23].

The CPTCI was shown to be highly correlated with scales measuring PTSD symptoms, depression, and anxiety. This may be due to the fact that PTSD symptoms are frequently accompanied by depression and anxiety, and it is consistent with findings from previous studies that showed high correlations between post-traumatic cognitions and depression and anxiety symptoms in children and adolescents [15, 17, 22, 45]. PTSD symptoms and CPTCI scores were significantly correlated even when depression and anxiety scores were controlled for, indicating that the correlation between these two sets of variables is not merely an artifact due to depression or anxiety, but rather due to cognitions and responses specific to traumatic experiences that are shared between the two sets of variables. Traumatic experiences are associated not only with PTSD, but also with various types of psychopathology, and it seems possible to examine post-traumatic cognitions as a transdiagnostic target of therapy and intervention [17, 19, 46, 47].

Earlier studies found that there were no significant differences in CPTCI results per age [15, 17]. However, we revealed the opposite. However, the sample characteristics may have affected our results. Previous studies reported that adolescent survivors of sexual violence are closely associated with more violent and severe assault characteristics like penetrative sexual assault, paid sex, brokering, and exhibit more serious and extensive psychological aftereffects than do child survivors [48, 49]. The adolescents included in the sample of this study also had a higher rate of exposure to rape rather than non-penetrative sexual harassment when compared to children, and their experiences were frequently accompanied by physical violence, multiple assailants, and so on. Other reasons for the CPTCI score differences per age may be related to cognitive and emotional development. In adolescence, more elaborate and complex emotions develop, and there is also the maturing of one’s self-concept and self-consciousness. Accordingly, one’s post-traumatic cognitions concerning threats to oneself, which are also one’s higher cognitions mediating secondary emotions, tend to become negatively distorted and exaggerated [48, 50–52]. Therefore, it is necessary to consider such age characteristics when interpreting the CPTCI results. In addition, future studies need to investigate whether CPTCI reveals any differences per age in the severity and persistence of maladaptation and psychological distress resulting from exposure to sexual violence. As for sex differences, which were not evident, the sample included few male survivors; therefore, it is difficult to interpret and generalize the research findings in this respect.

We also sought to verify the reliability and validity of the CPTCI-S. It was confirmed that the internal consistency, convergent validity, and discriminant validity of the CPTCI-S were similar to those of the CPTCI’s total score. Moreover, our confirmatory factor analysis showed that CPTCI-S had better overall model fit than the original 25-item scale, which was consistent with previous findings [21]. Among the fit indices for the CPTCI-S, the RMSEA did not have a good fit; however, this may be because the index in question has the property of yielding poor fit when there are only a few items or measurement variables and consequently few degrees of freedom [53]. When other indices such as the CFI, TLI, and SRMR were considered, they support the two-factor structure. Consequently, the CPTCI-S is expected to be useful in clinical practice and its subscales seem amenable to interpretation.

This study had some limitations. First, instead of using structured interviews with clinicians to perform PTSD diagnoses, the cutoff point for the self-report CRIES was used to distinguish the high PTSD-risk group and the low PTSD-risk group. The Korean version of the CRIES was found to have high sensitivity (.88) and specificity (.85; [35]); therefore, we felt it could be used to diagnose PTSD with relative accuracy. However, it is necessary to confirm the validity of the CPTCI through more precise criteria in the future. Second, formal backward translation has not been done. Third, this study was conducted only on survivors of sexual violence; therefore, it is difficult to generalize our results to groups exposed to other types of trauma. However, it must be made clear that this limitation is at the same time a strength of this study. Previous studies have shown that CPTCI scores and its factor structure may vary per type of trauma [15]. For this reason, the original CPTCI paper mentioned the need to apply the scale to various types of samples. Until now, however, no studies had confirmed the psychometric properties of CPTCI as applied solely to survivors of sexual violence. Another limitation is gross underrepresentation of males in the sample. Due to nature of the sexual violence, the sample consists mostly of females. Further study is needed to identify the characteristics of male survivors of the sexual assault.

Despite these limitations, this study is the first to use the CPTCI on child and adolescent survivors of sexual violence, thereby adding new evidence on the scale’s applicability. The present study may extend our understanding of the CPTCI by validating the scale in a different cultural context to previous studies, and in a homogenous sample regarding types of trauma. Further research should be undertaken to investigate the utility of the CPTCI and distinct response patterns considering types of trauma, the phases of response to trauma, and cultural differences.

## Conclusion

This study investigated the psychometric properties of the CPTCI among child and adolescent survivors of sexual violence in Korea. In general, the scale was found to be a valid instrument for measuring dysfunctional trauma-related cognitions. Moreover, the CPTCI-S was also confirmed to have excellent psychometric properties. Therefore, the Korean versions of the CPTCI and CPTCI-S are valuable tools that can be used in clinical and research settings to better understand the psychological mechanisms behind the responses of children and adolescents who have been exposed to trauma.

## Abbreviations

CPTCI: Child Post-Traumatic Cognitions Inventory
PTSD: post-traumatic stress disorder
CRIES: Children’s Revised Impact of Event Scale
TSCC: Traumatic Symptom Checklist for Children
CAPS: Children’s Attributions and Perceptions Scale
CDI: Children’s Depression Inventory
RCMAS: Revised Children’s Manifest Anxiety Scale
CPTCI-PC: permanent and disturbing change subscale of the CPTCI
CPTCI-SW: fragile person in a scary world subscale of the CPTCI
CFI: comparative fit index
TLI: Tucker–Lewis index
RMSEA: root mean square error of approximation
CI: confidence interval
SRMR: standardized root mean square error of approximation
